# Effectiveness of Immune Checkpoint Inhibitors in Transplant Recipients with Progressive Multifocal Leukoencephalopathy 

**DOI:** 10.3201/eid2511.190705

**Published:** 2019-11

**Authors:** Chloé Medrano, François Vergez, Catherine Mengelle, Stanislas Faguer, Nassim Kamar, Arnaud Del Bello

**Affiliations:** Hôpital Rangueil, Toulouse, France (C. Medrano, S. Faguer, N. Kamar, A. Del Bello);; Université Paul Sabatier, Toulouse (C. Medrano, F. Vergez, S. Faguer, N. Kamar, A. Del Bello);; Hôpital de Toulouse, Toulouse (F. Vergez, C. Mengelle);; Hôpital Purpan, Toulouse (C. Mengelle, N. Kamar, A. Del Bello)

**Keywords:** progressive multifocal leukoencephalopathy, PML, immune checkpoint inhibitors, nivolumab, T-cell exhaustion, kidney transplantation, JC virus, viruses, BK virus, immunosuppression

## Abstract

Antibodies against PD1 have been used to treat progressive multifocal leukoencephalopathy (PML), a rare brain disease caused by JC virus. We used these antibodies (nivolumab) to treat PML in 3 kidney transplant recipients. All died within 8 weeks of diagnosis. Hence, nivolumab did not improve PML outcome after solid organ transplantation.

The role of T-cell exhaustion in the development of progressive multifocal leukoencephalopathy (PML), a rare brain disease caused by JC virus, has prompted clinicians to use immune checkpoint inhibitor molecules to treat JC virus–infected patients. Recently, Cortese et al. (*1*) used antibodies against PD1 to treat PML in 8 patients (6 with a history of blood disorders and 2 with HIV infection). They noted improvement or stabilization of symptoms for 5 patients but no benefit for the others.

Since 2017, we have treated PML in 3 kidney transplant recipients with a definitive diagnosis, according to the American Academy of Neurology (https://www.aan.com) consensus, made 5 (range 2–17) years after transplantation. We have compiled clinical and radiologic findings for these patients (Appendix Figures 1–3). Since transplantation, the patients had been receiving mycophenolic acid and steroids with either belatacept (n = 1) or tacrolimus (n = 2). At PML diagnosis, immunosuppressants were immediately withdrawn, and nivolumab (antibodies against PD1) was given at a dose of 3 mg/kg every 15 days (2 injections for 2 patients and 3 injections for 1) (Table). For the patient who had received belatacept, we performed 3 apheresis sessions to remove the drug before nivolumab initiation. All patients died within the first 8 weeks after PML diagnosis because of rapid progression of neurologic symptoms. 

**Table:** Characteristics of 3 patients with PML who received nivolumab, France, 2017*

Patient characteristics	Total lymphocytes; CD4+; CD8+, n/mm^3^	Clinical course	Additional therapy	*JCV in CSF, log*_10_ copies/mL	Loss of kidney function
Patient 1: age 81 y; received transplant 5 y before PML diagnosis; received treatment with Tac, MPA, prednisone	B: 300; 76; 56/LFU: 1,000; 602; 250†	Rapid progression of neurologic disorders despite 2 injections of nivolumab; death from progression of PML 6 wk after diagnosis	Mirtazapine 15 mg/d	B: 3.5/LFU: NA	No
Patient 2: age 77 y; received transplant 2 y before PML diagnosis; received treatment with belatacept, MPA, and prednisone	B: 377; 162; 106/LFU: 444; 117; 210‡	Rapid progression of neurologic disorders despite 3 injections of nivolumab; death from progression of PML 6 wk after diagnosis	Mirtazapine 15 mg/d; γ interferon therapy (100 μg) added 1 day after second and third injections	B: 2.9/LFU: 5	Yes
Patient 3: age 67 y; received transplant 17 y before PML diagnosis; received treatment with Tac, MPA, prednisone	B: 487; 287; 67/LFU: 2,076; 1,183; 477§	Rapid neurologic degradation despite 2 injections of nivolumab; death from progression of PML 4 wk after diagnosis	Mirtazapine 15 mg/d	B: 2.9/LFU: NA	No

*B, baseline; CSF, cerebrospinal fluid; JCV, JC virus; LFU, last follow-up; MPA, mycophenolic acid; NA, not available, PML, progressive multifocal leukoencephalopathy; Tac, tacrolimus. †LFU for patient 1 was 1 wk after the second injection of nivolumab. ‡LFU for patient 2 was 4 d after the third injection of nivolumab. *§*LFU for patient 3 was 1 wk after the second injection of nivolumab.

Magnetic resonance imaging was performed before each injection and a few days before death, but images showed no signs of immune reconstitution inflammatory syndrome. Conversely, images did show progression of PML features. As expected, the percentage of T cells expressing PD1, which was assessed for 2 patients, dramatically decreased after receipt of nivolumab (Appendix Figure 4), whereas other inhibitory receptors tested (2b4 and CD160) remained stable or increased. In addition, functional analysis showed a reduction of cytokine production by CD4+ and CD8+ T cells and an improvement of cytotoxic ability, a phenotype compatible with more terminally differentiated exhausted cells, which are less likely to respond to anti-PD1 immune checkpoint inhibitor (*2*).

Research has suggested that PML could occur at any time after transplantation (*3*), even several years after engraftment, which was the case for the 3 patients reported here. As opposed to the results reported by Cortese et al. (*1*), the outcomes for the 3 patients we report, who received nivolumab, was very bad and in line with the PML outcomes usually reported after solid-organ transplant patients (i.e., median survival time <6 months) (*3*). The difference between the patients reported by Cortese et al. and the patients that we report is probably use of immunosuppressive agents (calcineurin inhibitors or costimulation blockers) that can lead to persistent T-cell dysfunction, despite withdrawal of these treatments, resulting in refractory T-cell dysfunction after use of anti-PD1 blockers, as reported in ex vivo experiments (*4*). This hypothesis is supported by the absence of kidney rejection in 2 of the 3 patients. Of note, all 5 patients reported by Cortese et al. (*1*) for whom anti-PD1 blockers were efficient were not receiving immunosuppressive therapy at PML diagnosis. 

Moreover, the 3 patients reported here had profound lymphopenia at diagnosis, which for 2 patients did not improve after receipt of nivolumab (Table). Although there is no established relationship between the severity of lymphopenia and the response to anti-PD1, the 3 patients with unfavorable outcomes reported by Cortese et al. (*1*) also had severe lymphopenia. This finding suggests that immunotherapies can be ineffective in patients with severe lymphopenia. The use of ex vivo expanded, BK virus–specific T cells (*5*) should be tested in this setting. For the kidney transplant patients with PML reported here, use of nivolumab, associated with immunosuppressive therapy withdrawal, did not restore efficient immune response and did not improve the outcomes. 

Appendix Clinical course of disease for 3 transplant recipients who received immune checkpoint inhibitors (nivolumab) for severe progressive multifocal leukoencephalopathy; expression of surface T-cell inhibitory molecules in 2 patients after receipt of nivolumab.

## References

[1] Cortese I, Muranski P, Enose-Akahata Y, Ha SK, Smith B, Monaco M, et al. Pembrolizumab treatment for progressive multifocal leukoencephalopathy. N Engl J Med. 2019;380:1597–605. 10.1056/NEJMoa1815039 30969503

[2] Blackburn SD, Shin H, Freeman GJ, Wherry EJ. Selective expansion of a subset of exhausted CD8 T cells by alphaPD-L1 blockade. Proc Natl Acad Sci U S A. 2008;105:15016–21. 10.1073/pnas.0801497105 18809920 PMC2567485

[3] Mateen FJ, Muralidharan R, Carone M, van de Beek D, Harrison DM, Aksamit AJ, et al. Progressive multifocal leukoencephalopathy in transplant recipients. Ann Neurol. 2011;70:305–22. 10.1002/ana.22408 21823157 PMC4910883

[4] Dekeyser M, de Goër de Herve MG, Hendel-Chavez H, Labeyrie C, Adams D, Nasser GA, et al. Refractory T-Cell anergy and rapidly fatal progressive multifocal leukoencephalopathy after prolonged CTLA4 therapy. Open Forum Infect Dis. 2017;4:ofx100. 10.1093/ofid/ofx100 28638849 PMC5473436

[5] Muftuoglu M, Olson A, Marin D, Ahmed S, Mulanovich V, Tummala S, et al. Allogeneic BK virus-specific t cells for progressive multifocal leukoencephalopathy. N Engl J Med. 2018;379:1443–51. 10.1056/NEJMoa1801540 30304652 PMC6283403

