# Supplementary material for: Effectiveness of Immune Checkpoint Inhibitors in Transplant Recipients with Progressive Multifocal Leukoencephalopathy
Source: Emerg Infect Dis. 2019 Nov;25(11):2145–7. doi: 10.3201/eid2511.190705 (PMC6810212; doi:10.3201/eid2511.190705)
Supplement: Appendix — Clinical course of disease for 3 transplant recipients who received immune checkpoint inhibitors (nivolumab) for severe progressive multifocal leukoencephalopathy; expression of surface T-cell inhibitory molecules in 2 patients after receipt of nivolumab. [file 19-0705-Techapp-s1.pdf]

Article DOI: https://doi.org/10.3201/eid2511.190705

Effectiveness of Immune Checkpoint Inhibitors in Transplant Recipients with Progressive Multifocal Leukoencephalopathy

Appendix

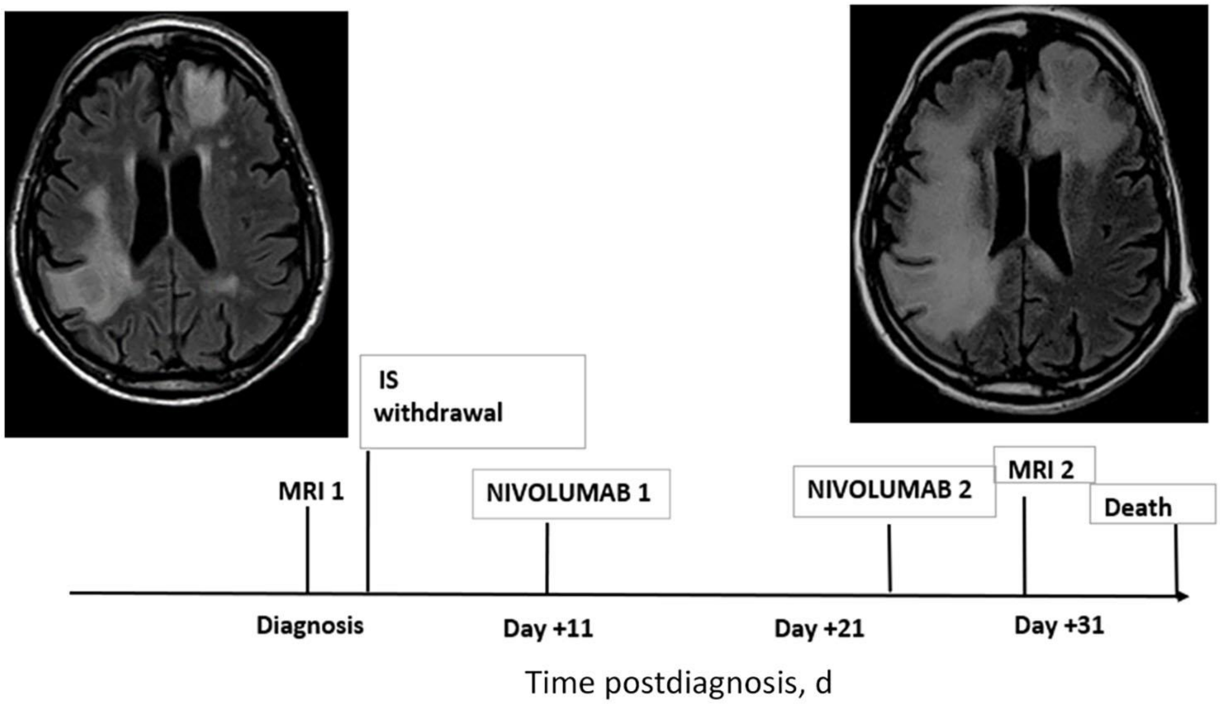

Appendix Figure 1. Clinical course of patient 1. An 81-year-old man had undergone a kidney transplantation in 2012 because of end-stage renal disease related to nephroangiosclerosis. He was given induction therapy by basiliximab followed by a triple immunosuppressive regimen including tacrolimus, mycophenolate mofetil, and prednisone. No acute rejection occurred after transplantation. Five years after kidney transplantation, the patient presented with behavioral disorders and confusion, without fever. At admission, brain-MRI showed several bilateral hyperintense lesions, involving left frontal and right parietal lobe. The diagnosis of PML was confirmed by the detection of JCV DNA in CSF (3.5

log-copies/mL). At day 11, because of worsening of neurologic symptoms (patient unable to sit, psychomotor retardation and somnolence), nivolumab was initiated, and repeated 2 weeks later. One month after the first symptoms, MRI sequences showed several bilateral confluent hyperintense lesions, extended to both frontal lobes, and right parietal lobe without evidence of an inflammatory reaction with edema, mass effect, or gadolinium enhancement suggesting the occurrence of immune reconstitution inflammatory syndrom. The patient became bed bound, felt into coma and died roughly 1 month and a half after admission.

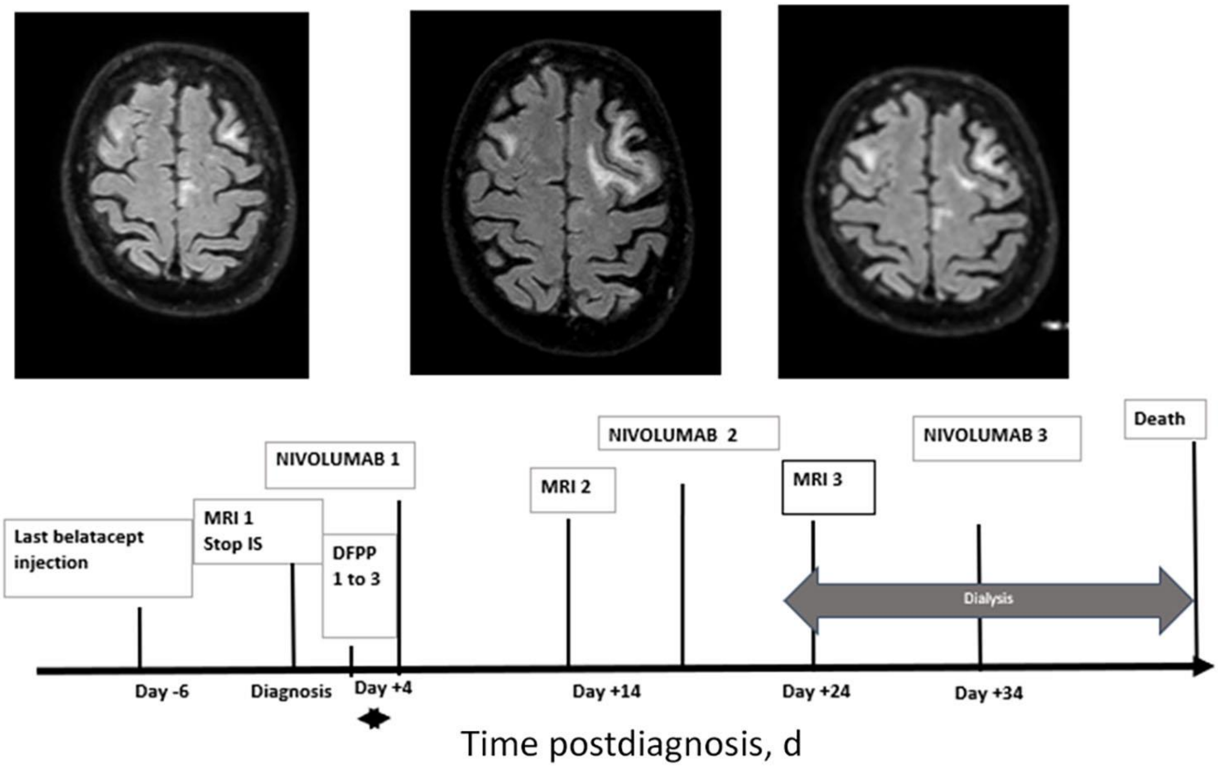

Appendix Figure 2. Clinical course of patient 2. A 77-year-old Caucasian man had undergone a kidney transplantation in 2015 because of nephroangiosclerosis and diabetic nephropathy. No induction therapy was given. Maintenance immunosuppression included tacrolimus, MMF, and prednisolone. One year after transplantation, the patient was converted from tacrolimus to belatacept because of biopsy-proven calcineurin inhibitors nephrotoxicity. At conversion, serum creatinine level was at 33 mg/L. Two years after transplantation (one year after belatacept initiation), the patient presented with profound weakness. At admission, brain FLAIR-MRI sequences revealed bilateral hyperintense lesions in both frontal lobes. The diagnosis of PML was confirmed by the detection of JCV DNA in CSF (2.9 log-copies/mL). Belatacept and MMF were stopped immediately after the diagnosis. In addition, the patient had undergone three sessions of double filtration plasmapheresis (DFPP) to eliminate rapidly belatacept. Immediately after the 3 sessions of DFPP, nivolumab was started (3mg/kg each 15 days). Gamma-

interferon (Intravenous perfusion of Imukin, 100 µg) was added to the second and third injection of nivolumab because of neurologic symptoms deterioration (somnolence, bed-ridden). The second and third MRI showed extension of lesions, without signs of IRIS. The patient died 1 month and half after admission. `

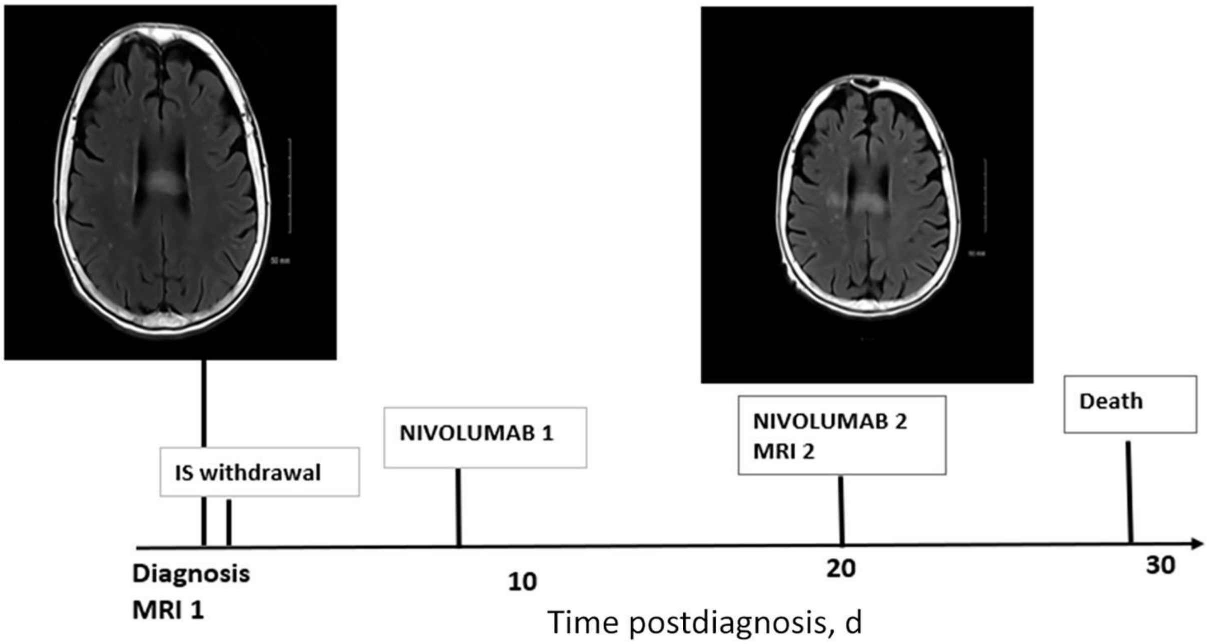

Appendix Figure 3. Clinical course of patient 3. A 67-years-old woman had undergone a kidney transplantation in 2007 due to interstitial nephropathy. She was given induction therapy by basiliximab, followed by a triple immunosuppressive therapy including belatacept, MMF, and prednisone. Eleven years after transplantation, belatacept was replaced by tacrolimus, for patient personal suitability. The postransplantation period was uneventful and no acute rejection occurred. Two months after the switch, the patient was admitted for left hemiparesis worsening for the past month. At admission, brain MRI showed PML-compatible bilateral lesions, with hyperintense lesions in corpus callusum in FLAIR sequences. The diagnosis of PML was confirmed by the detection of JCV DNA in CSF (2.9 log-copies/mL). Tacrolimus and MMF were stopped immediately after the diagnosis. Prednisolone was maintained, and mirtazapine was added (15mg/d). Nivolumab (3mg/kg) was started immediately. Because of rapid neurologic symptoms impairment 1 week after the first injection (bed-ridden, somnolence), the patient was given a second injection of nivolumab (3 mg/kg). A brain MRI performed 1 week after the second injection of nivolumab, confirmed the PML progression without argument for IRIS. The patient presented went into coma and died 4 weeks after admission.

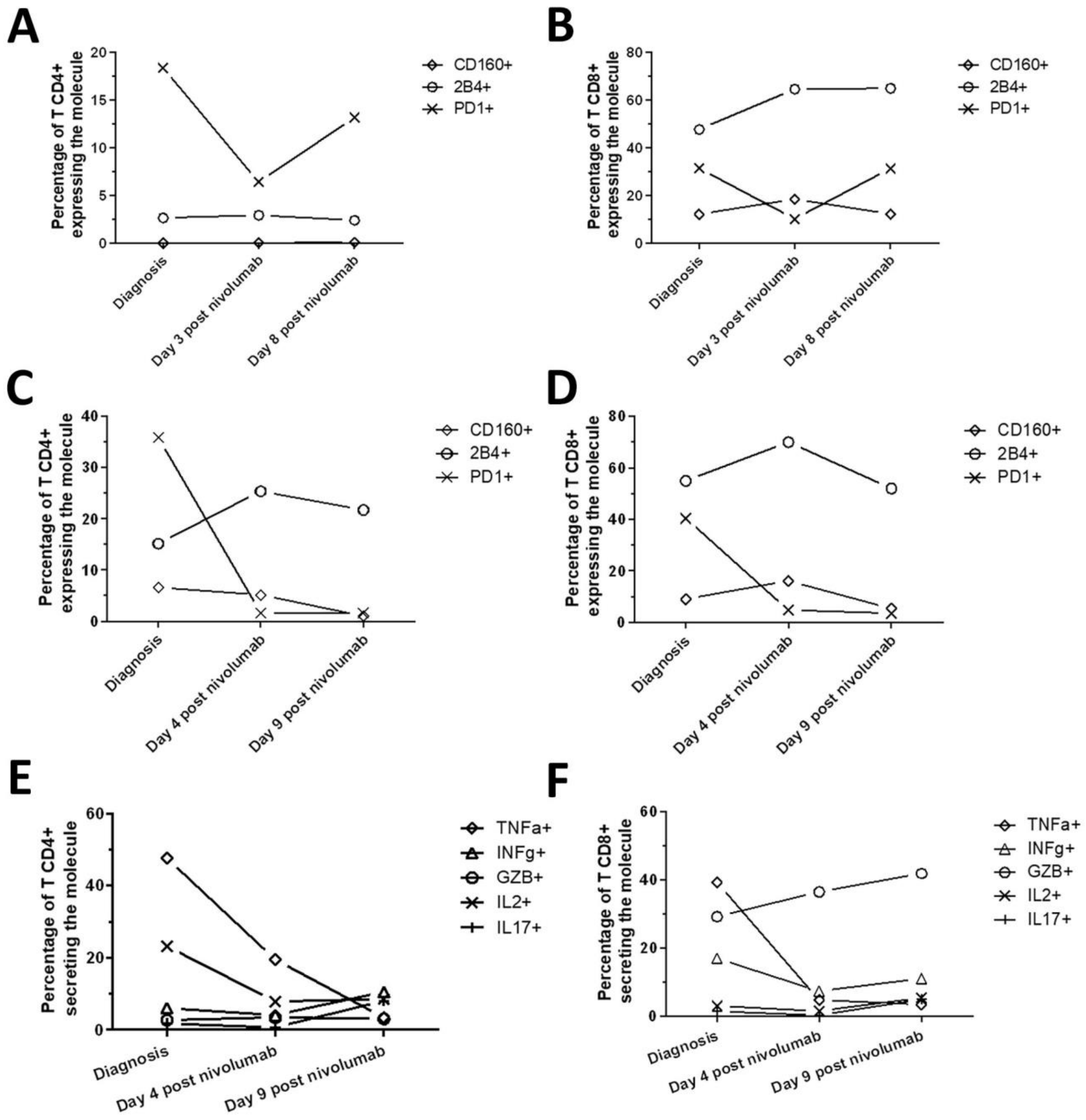

Appendix Figure 4. Expression of surface T cells inhibitory molecules in Patient 2 and 3 at diagnosis and after nivolumab injection. Phenotypic analysis of T-cells inhibitory molecules (PD-1, 2B4, CD160) expression in CD4+ (A) and CD8+ (B) cells before and after nivolumab injection in patient 2. Phenotypic analysis of T-cells inhibitory molecules (PD-1/2b4/CD160) in CD4+ (C) and CD8+ (D) cells before and after nivolumab injection in patient 3. In vitro CD4+ (E) and CD8+ (F) T cells production of TNFα, IL-2, IFNγ, Granzyme B (GZB) and IL-17, after unspecific stimulation with PMA/ionomycin, before and after nivolumab injection in patient 3. 50 µl of fresh heparinized blood were incubated for 3h at 37°C, 5% CO2 in a Duractive 1 tube (Beckman Coulter), containing a fixed amount of PMA and Ionomycin. Fixation,

permeabilization and intracellular stainings were performed with the Perfix NC kit and the Duraclone IFT activation kit (Beckman Coulter), according to the manufacturer's instructions. Following the procedure, cells were immediately analyzed by flow cytometry.
